# Vitamin A Transport Mechanism of the Multitransmembrane Cell-Surface Receptor STRA6

**DOI:** 10.3390/membranes5030425

**Published:** 2015-08-28

**Authors:** Riki Kawaguchi, Ming Zhong, Miki Kassai, Mariam Ter-Stepanian, Hui Sun

**Affiliations:** Department of Physiology, Jules Stein Eye Institute, and Howard Hughes Medical Institute, David Geffen School of Medicine, University of California, Los Angeles, CA 90095, USA

**Keywords:** vitamin A, STRA6, plasma retinol binding protein, membrane transport mechanism, RBP, retinoid, vision, cancer, skin disease, fenretinide

## Abstract

Vitamin A has biological functions as diverse as sensing light for vision, regulating stem cell differentiation, maintaining epithelial integrity, promoting immune competency, regulating learning and memory, and acting as a key developmental morphogen. Vitamin A derivatives have also been used in treating human diseases. If vitamin A is considered a drug that everyone needs to take to survive, evolution has come up with a natural drug delivery system that combines sustained release with precise and controlled delivery to the cells or tissues that depend on it. This “drug delivery system” is mediated by plasma retinol binding protein (RBP), the principle and specific vitamin A carrier protein in the blood, and STRA6, the cell-surface receptor for RBP that mediates cellular vitamin A uptake. The mechanism by which the RBP receptor absorbs vitamin A from the blood is distinct from other known cellular uptake mechanisms. This review summarizes recent progress in elucidating the fundamental molecular mechanism mediated by the RBP receptor and multiple newly discovered catalytic activities of this receptor, and compares this transport system with retinoid transport independent of RBP/STRA6. How to target this new type of transmembrane receptor using small molecules in treating diseases is also discussed.

## 1. Introduction

An organism can obtain difficult-to-synthesize organic compounds from the environment instead of doing *de novo* synthesis. All vitamins are organic compounds that fall into this category and epitomize the intimate dependence of organisms on the environment for survival. Vitamins have been given alphabetical names starting with vitamin A. Vitamin A is arguably the most multifunctional vitamin in the human body and is essential for human survival at every point from embryogenesis to adulthood. The diversity of biological functions of vitamin A and its derivatives is astonishing and is still not fully known. The chemical basis of this versatility is the transformation of vitamin A into a group of related compounds, known as retinoids, that differ in their configuration of double bonds, their oxidation state, and other modifications. The biological functions of retinoids affect every human organ from embryogenesis to adulthood and are still being discovered. The aldehyde form of vitamin A functions as the chromophore for visual pigments in the eye [1,2,3,4,5] and also regulates adipogenesis [6]. The acid form of vitamin A (retinoic acid) has the most diverse functions [7]. Nuclear retinoic acid receptors regulate the transcription of a large number of genes [8,9]. In addition to its essential roles in embryonic development [10,11], retinoic acid is also important in the function of many adult organs such as the nervous system [12,13], the immune system [14,15], the male and female reproductive systems [16,17], the respiratory system [18,19], and the skin [20]. Retinoids have also been used successfully as therapeutic agents in treating human diseases including leukemia and acne. A combination of retinoic acid and arsenic trioxide offered the first successful cure of a specific type of cancer: acute promyelocytic leukemia [21]. 

If we depend on the environment to obtain such an important molecule that is absolutely essential for survival, how can the body guarantee a stable supply when there is insufficient vitamin A available in food? Like any chemical drug that has potent biological activities, vitamin A —and its derivatives— can have strong side effects if it acts at the wrong place, the wrong time or the wrong dosage [22]. Since excessive vitamin A is toxic, how can we make sure that the delivery mechanism achieves the precise amount? Evolution came up with a specific and dedicated vitamin A transport mechanism mediated by plasma retinol binding protein (RBP), the principal means of vitamin A transport in the blood [23,24,25,26,27,28,29]. Virtually all vitamin A in the blood under physiological conditions is bound to RBP. The choice of retinol as the main transport form of vitamin A makes biological sense. Retinol is one of the least toxic forms of vitamin A and can also serve as the precursor to other biologically active forms. 

## 2. The Membrane Receptor that Mediates Cellular Uptake of Vitamin A from the Blood

**Figure 1:**
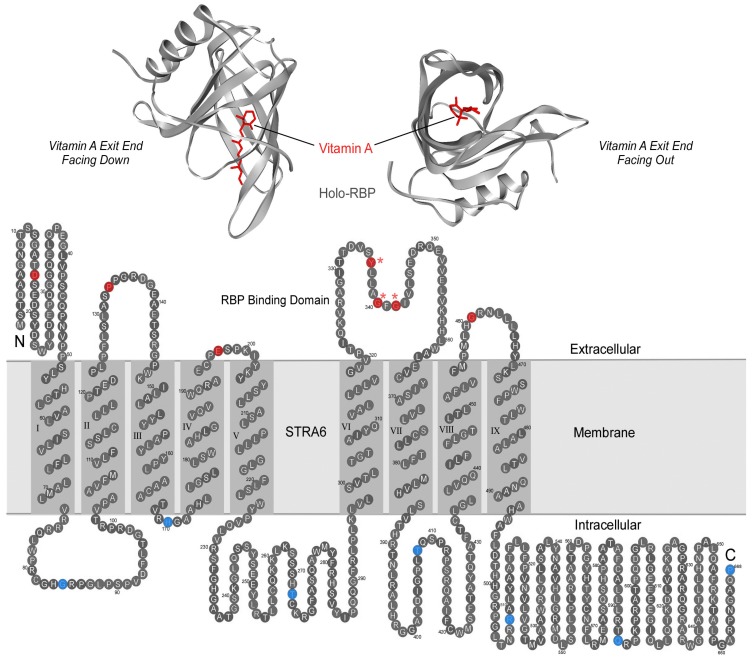
Plasma retinol binding protein and its transmembrane receptor STRA6. **A.** Crystal structures of holo-RBP with the vitamin A exit end facing down (left picture) or facing out (right picture). **B.** The transmembrane topology model of STRA6. Residues that have been experimentally confirmed to be extracellular are labeled in red. Residues that have been experimentally confirmed to be intracellular are labeled in blue. The three STRA6 residues essential for RBP binding are labeled with asterisks. A mutation in either one of these three residues is sufficient to abolish RBP binding and vitamin A uptake without affecting STRA6’s cell-surface expression.

In the mid-1970s, it was proposed that there exists a membrane receptor for RBP to take up vitamin A from RBP [30,31,32]. Using an unbiased strategy combined with mass spectrometry, this receptor was identified as STRA6, a multitransmembrane protein of previously unknown function [33,34]. This receptor represented both a new class of membrane transport protein and a new class of membrane receptor. Even its transmembrane topology was unknown at the time of its discovery. Systematic structural analyses determined that STRA6 has nine transmembrane domains, five extracellular domains and four intracellular domains [35] (Figure 1). Consistently, a large-scale unbiased mutagenesis study identified an essential RBP binding domain located between transmembrane domain VI and VII (Figure 1) [36]. How does STRA6 take up vitamin A from holo-RBP? 

**Figure 2:**
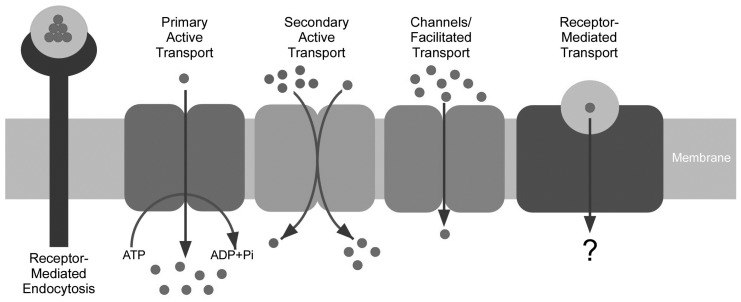
Comparison of STRA6-mediated vitamin A uptake from holo-RBP (right diagram) with known cellular uptake mechanisms including receptor-mediated endocytosis, primary active transport, secondary activity transport, channels and facilitated transport.

Cells are known to take up extracellular molecules through receptor-mediated endocytosis, primary active transport, secondary active transport, channels and facilitated transport (Figure 2). STRA6-mediated vitamin A uptake is not an example of primary active transport because STRA6 has no ATP binding domain. The fact that metabolic inhibitors do not stop the uptake process, and that the uptake happens efficiently in a cell-free system without any energy source [33] argues against any mechanism that depends on cellular energy. The only remaining possibility is channel/facilitative transport, which depends on the electrochemical gradient of the free ligand. However, STRA6’s transport substrate is not free but is bound with high affinity to its binding protein to form a 1:1 complex (the definition of holo-RBP). Independent studies have shown that STRA6 does not facilitate the uptake of free retinol by LRAT [37] or CRBP-I [38]. Therefore, STRA6’s mechanism is distinct from all known cellular uptake mechanisms (Figure 2). 

## 3. STRA6’s Vitamin A Uptake Mechanism and Its Coupling to Intracellular Proteins

**Figure 3:**
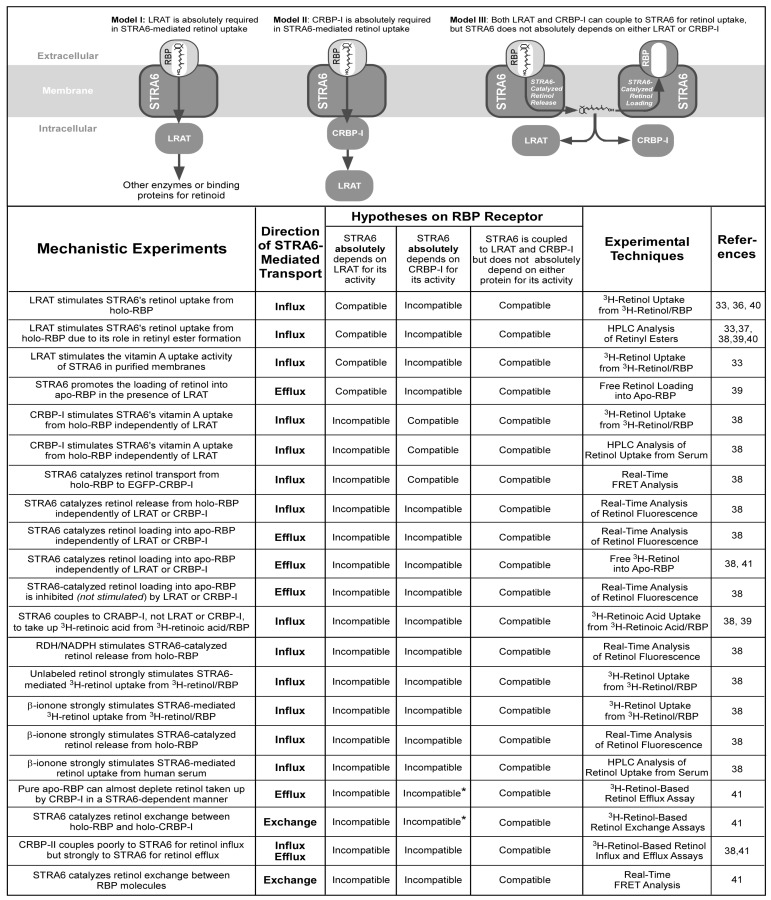
Major hypotheses on the mechanism of STRA6-mediated cellular vitamin A uptake from holo-RBP and experimental evidence that is compatible or incompatible with these hypotheses. Evidence revealed by diverse mechanistic experiments argues against the absolute dependence of STRA6 on either LRAT or CRBP-I. In the STRA6 activities marked by asterisks, STRA6 catalyzes the removal of retinol from CRBP-I. These activities are also incompatible with models that proposed the absolute requirement of CRBP-I for STRA6’s activity because these models also proposed that only apo-CRBP-I interacts with STRA6.

As soon as STRA6 was identified as the RBP receptor, we [33,38] and other investigators [37,39,40] found that LRAT stimulates STRA6’s vitamin A uptake activity from holo-RBP. We found that LRAT stimulates STRA6’s activity as effectively in live-cell assays as in a cell-free system that consists of only purified membranes [33]. LRAT’s role in vitamin A uptake was studied in detail by Amengual and colleagues [40]. We also demonstrated the stimulation of STRA6’s vitamin A uptake activity by CRBP-I [38]. Given the roles of LRAT and CRBP-I in stimulating STRA6’s activity, there are three major models for STRA6’s vitamin A uptake mechanism (Figure 3). In model I, STRA6 absolutely depends on LRAT for its activity. This model might explain the large stimulatory effect of LRAT on STRA6’s vitamin A uptake activity. In this model, retinol converted by LRAT to retinyl ester is subsequently delivered to other retinoid processing enzymes or retinoid binding proteins. In model II, STRA6 absolutely depends on CRBP-I for its activity. Retinol taken up by CRBP-I is subsequently delivered to other retinoid processing enzymes such as LRAT. This model may explain the stimulatory effect of CRBP-I on STRA6’s vitamin A uptake activity. In model III, although STRA6 couples to LRAT or CRBP-I for vitamin A uptake, neither protein is absolutely required for its activity [38,41]. 

Despite the abilities of model I or model II to explain a few experimental results, model III is the correct model (Figure 3). Evidence against model I includes the ability of CRBP-I to couple to STRA6 for retinol uptake, the ability of CRABP-I to couple to STRA6 for retinoic acid uptake, the inability of LRAT to couple to STRA6 for retinoic acid uptake, and the ability of β-ionone to stimulate STRA6-mediated vitamin A uptake independent of LRAT, as discussed in detail below [38]. In addition, LRAT’s intracellular localization to smooth ER [42] makes it unlikely to physically interact with STRA6 located on the cell surface. Evidence against model II includes the ability of LRAT to couple to STRA6 for retinol uptake independently of CRBP-I, the ability of CRABP-I to couple to STRA6 for retinoic acid uptake, the inability of CRBP-I to couple to STRA6 for retinoic acid uptake, and the ability of β-ionone to stimulate STRA6-mediate vitamin A uptake independent of CRBP-I [38]. 

Overwhelming evidence provided by diverse mechanistic experiments using many techniques supports model III (Figure 3). STRA6 has many LRAT or CRBP-I independent activities, and many of these activities are suppressed, not enhanced, by the presence of either CRBP-I or LRAT [38,41]. STRA6 catalyzes retinol release from holo-RBP independently of LRAT and CRBP-I. STRA6 also catalyzes retinol loading into apo-RBP independently of LRAT and CRBP-I. STRA6 not only does not depend on LRAT or CRBP-I for this activity, LRAT or CRBP-I actually inhibits this STRA6 activity. STRA6-catalyzed retinol release from holo-RBP and STRA6-catalyzed retinol loading into apo-RBP largely cancel each other’s action. Anything that inhibits STRA6-catalyzed retinol loading stimulates STRA6-catalyzed retinol release. LRAT or CRBP-I enhances STRA6-catalyzed retinol release from holo-RBP indirectly by removing the substrate of the loading reaction, *i.e.*, retinol. Even small molecules can stimulate STRA6’s vitamin A uptake like LRAT or CRBP-I, if the molecule can inhibit STRA6-catalyzed retinol loading. For example, unlabeled retinol potently stimulates STRA6-mediated ^3^H-retinol uptake from ^3^H-retinol/RBP [38]. Free retinol’s ability to stimulate STRA6’s uptake of ^3^H-retinol from ^3^H-retinol/RBP is consistent with model III because unlabeled retinol competes with ^3^H-retinol in the loading reaction. β-ionone, which mimics retinol in structure but is not a retinoid, can stimulate STRA6’s retinol release activity in all assays by inhibiting the loading reaction (not just the radioactive assay) [38]. 

In summary, STRA6’s catalytic activities including retinol release from holo-RBP and retinol loading into apo-RBP can explain its role as the RBP receptor in mediating vitamin A uptake and its coupling to intracellular proteins involved in vitamin A storage (CRBP-1 and LRAT). This is likely achieved by opening up the vitamin A exit end of RBP to allow retinol to come in or out to promote these reversible reactions. These two reversible activities cancel each other’s action so that no retinol is released into the cell unless there is a way to store retinol. CRBP-I or LRAT couples to STRA6 in vitamin A uptake because they inhibit the retinol loading reaction by removing retinol. Similarly, small molecules like ß-ionone can also stimulate STRA6’s retinol release activity by inhibiting its retinol loading reaction. 

## 4. STRA6-Catalyzed Vitamin A Influx, Efflux and Exchange

**Figure 4:**
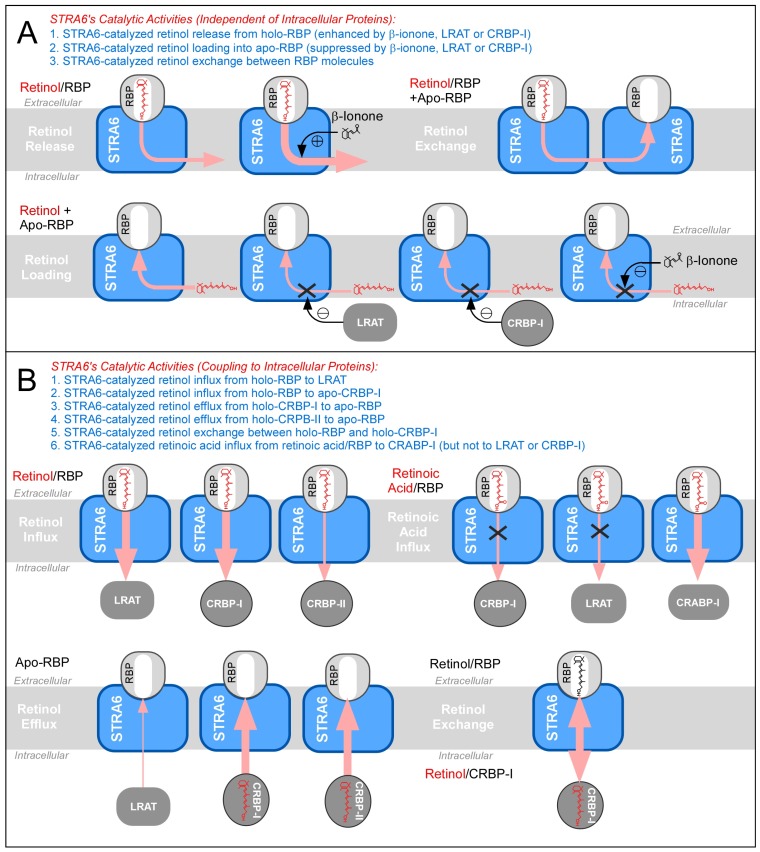
Schematic diagrams of the diverse catalytic activities of STRA6. (**A**) Summary of STRA6’s catalytic activities independent of intracellular proteins and the influences (stimulation or inhibition) of intracellular proteins on these activities. (**B**) Summary of STRA6’s activities that couple to intracellular proteins. The directions of retinol transport are indicated by the light red arrows. The efficiency of retinol transport is indicated by the thickness of each arrow.

Recent mechanistic studies have revealed the diverse catalytic activities of STRA6 (Figure 4) and these activities strongly support model III and argue against model I and II. Ultimately, all these activities can be explained by STRA6’s ability to reversibly promote retinol release from holo-RBP or retinol loading into apo-RBP. In addition to promoting cellular vitamin A uptake (retinol influx), STRA6 can also promote retinol efflux [39,41], retinol exchange between extracellular RBP and intracellular CRBP-I [41] and retinol exchange between RBP molecules [41]. Exchange means that retinol in one binding protein (e.g., RBP) can exchange retinol with another binding protein (e.g., CRBP-I) with no net gain or loss of retinol on either protein. 

**Figure 5:**
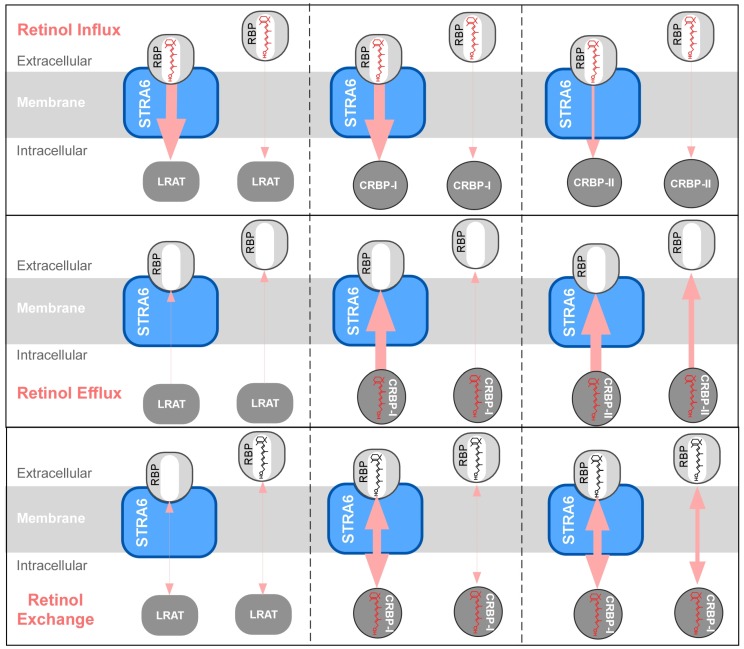
Schematic diagrams comparing the activities of LRAT, CRBP-I, and CRBP-II in STRA6-dependent or STRA6-independent retinol influx, efflux, and exchange. In retinol exchange diagrams, intracellular and extracellular retinol molecules are drawn in different colors (for diagrams involving LRAT, retinyl ester is not drawn). The directions of retinol transport are indicated by the light red arrows. The efficiency of retinol transport is indicated by the thickness of each arrow.

Since retinol influx and retinol efflux are opposing activities, it is important to understand the extracellular and intracellular factors that determine the direction of retinol transport in STRA6 (Figure 5). In retinol influx from holo-RBP, STRA6 couples effectively to LRAT and CRBP-I, but poorly to CRBP-II [38]. In retinol efflux into apo-RBP, STRA6 couples effectively to CRBP-I and CRBP-II, but poorly to LRAT. Pure apo-RBP can completely deplete of retinol taken up by CRBP-I in a STRA6-dependent manner. However, natural blood contains both holo-RBP and apo-RBP. When STRA6 encounters both holo-RBP and apo-RBP, holo-RBP blocks STRA6-mediated retinol efflux by competing with apo-RBP’s binding to STRA6 and by counteracting retinol efflux with influx [41]. In STRA6-catalyzed exchange between extracellular holo-RBP and intracellular retinoid pool, STRA6 again couples effectively to CRBP-I and CRBP-II, but poorly to LRAT. Although the function of STRA6’s retinol exchange activity between intracellular CRBP-I and extracellular RBP is not known, it may serve to refresh the intracellular retinoid pool.

The catalytic activities of STRA6 can explain the paradox of intriguing findings from several early studies. First, although the liver is responsible for most of holo-RBP secretion, an early study revealed that 50%–70% of the total input of retinol into the circulation comes from extrahepatic tissues [41]. STRA6’s potent retinol exchange activity between intracellular CRBP-I and extracellular RBP likely contributes to retinol input from extrahepatic tissues. Second, muscle-expressed human RBP can rescue the vision defect in RBP null mice [43], although muscle-expressed human RBP cannot mobilize liver-stored vitamin A [44]. STRA6’s retinol efflux activity from intracellular CRBP-I to human RBP is likely responsible for the retinol in human RBP (apo-RBP secreted by the muscle) that rescues the vision defect. Third, an early study that loaded the retinal pigment epithelium (RPE) cell with free ^3^H-retinol observed unexpectedly strong ^3^H-retinol efflux into the blood [45]. Because STRA6-catalyzed retinol exchange between intracellular and extracellular retinol binding proteins is not inhibited by the presence of holo-RBP in the blood, ^3^H-retinol efflux from the RPE into the blood is also likely due to this activity of STRA6. 

## 5. Isomer-Specific Effects of Retinoids on STRA6 Activity

**Figure 6:**
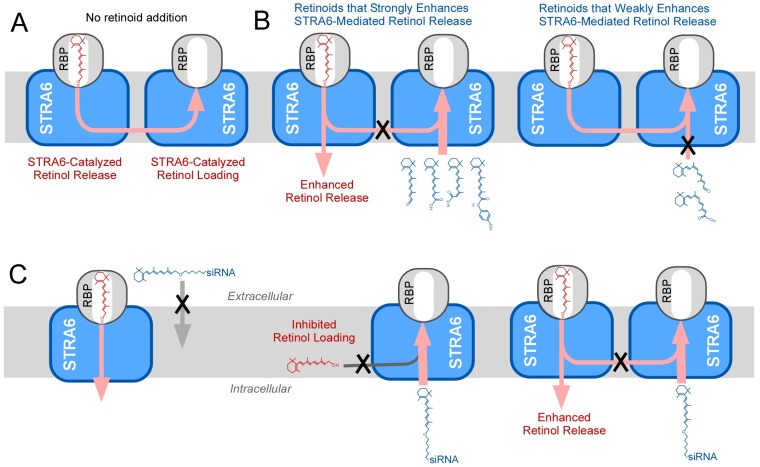
Schematic diagrams of the effects of retinoids on STRA6’s activities. **A.** The key activities of STRA6 are to catalyze retinol loading into apo-RBP and retinol release from holo-RBP. **B.** Fenretinide, all-trans retinal, all-trans retinoic acid and 13-cis retinoic acid more effectively stimulates STRA6-mediated retinol release and inhibits STRA6-mediated retinol loading than 9-cis retinol, 9-cis retinal and 9-cis retinoic acid. **C.** Schematic diagram of the effects of retinol-siRNA on STRA6’s activity. When added extracellularly, retinol-siRNA has no effect on STRA6-mediated vitamin A uptake from holo-RBP. However, retinol-siRNA potently stimulates STRA6-mediated retinol release and suppresses STRA6-mediated retinol loading when added to membranes that express STRA6.

How retinoids interact with RBP has been extensively studied since more than 40 years ago [46,47,48,49], but the effects of retinoids on the RBP receptor were unknown before the receptor’s identification due to the complications from endogenous retinoid binding proteins and related enzymes. In a recent study using both natural and artificial retinoids, the differential and isomer-specific interactions between retinoids and the RBP receptor were investigated [50]. This study offered unique insights into the receptor’s substrate uptake mechanism. The first finding is that certain retinoids can have profound effects on STRA6’s catalytic activities. For example, fenretinide can completely suppress STRA6’s vitamin A loading activity and strongly stimulate STRA6-catalyzed retinol release from holo-RBP. The second major finding is that effects of retinoids are highly isomer specific (Figure 6A and Figure 6B). All-trans isomers tend to be much more potent than 9-cis isomers in stimulating STRA6’s vitamin A uptake activity. Since 9-cis retinoid does bind RBP [47,51], the inability of 9-cis retinoid to stimulate STRA6-mediated vitamin A uptake is likely due to their inability to pass through STRA6 effectively (Figure 6B). The isomer-specific effects of retinoid’s action demonstrate the high specificity in the interaction of retinoids with the vitamin A uptake machinery. The third finding is that STRA6 highly enhances the ability of certain retinoids to release retinol from holo-RBP. This activity is likely explained by STRA6’s ability to open up the vitamin A exit end of RBP to allow retinol to come in or out. For example, fenretinide is more potent in causing retinol release from holo-RBP in the presence of STRA6 than in the absence of STRA6. The role of STRA6 in causing retinol release by fenretinide was also elegantly demonstrated in a recent study by Amengual and colleagues using STRA6 knockout mouse model [52]. They found that fenretinide decreases serum RBP in a STRA6-dependent manner, consistent with the role of STRA6 in enabling fenretinide’s retinol release from RBP. A related recent finding is that knocking down STRA6 expression decreases the effect of fenretinide on cancer cells [53]. 

In addition, experiments using a retinol-siRNA conjugate (siRNA covalently attached to the alcohol end of retinol) provided unique structural insights into STRA6/retinoid interaction (Figure 6C). First, retinol is oriented with the β-ionone ring end first when it is loaded into RBP by STRA6. Like other all-trans retinoids, retinol-siRNA stimulates STRA6’s retinol release and inhibits STRA6’s retinol loading [50]. Compared to retinol itself, retinol-siRNA has an intact β-ionone ring but a modified alcohol end. The fact that the very long conjugation at the alcohol end of retinol-siRNA does not prevent its effect on STRA6 is a clear demonstration the β-ionone ring end enters first when it is loaded into RBP through STRA6. Second, this unique reagent provides clues as to the orientation of STRA6 in interacting with retinoids. The long soluble and membrane-impermeable siRNA tail of the retinol-siRNA conjugate does not interfere with its ability to act on RBP/STRA6 when STRA6 is in the membrane form (which allows free access to both intracellular and extracellular sides of STRA6), but does prevent its ability to affect STRA6-mediated vitamin A uptake in the live cell assay. The fact that membrane impermeable retinol-siRNA can only affect RBP/STRA6 from the cytoplasmic side is consistent with the interaction of retinoids with the RBP/STRA6 complex through a pore facing into the cell (Figure 6C). 

## 6. STRA6 as a Therapeutic Target for Small Molecule-Based Drugs

A major finding on STRA6 mechanism is that many chemical compounds with the β-ionone ring or even the tiny β-ionone itself can potently stimulate STRA6-mediated vitamin A uptake [38]. Given the specific and dose-dependent nature of β-ionone’s effect, β-ionone or related compounds can be potentially employed to treat human diseases caused by insufficient tissue retinoid levels [13,20,54]. Systemic retinoid therapy has been used in treating human diseases in dermatology and oncology but is associated with serious side effects [55,56,57,58]. The advantage of the new strategy is that it stimulates a natural cellular vitamin A uptake mechanism in cells that naturally take up vitamin A using chemicals that are not necessarily retinoid (e.g., β-ionone). Since certain cancer cells are known to have more than 100 fold higher STRA6 expression levels [59], this strategy may also be used to target cancer cells. STRA6 was first known as a cancer cell surface marker before it was known as the RBP receptor. Interestingly, β-ionone is known to suppress carcinogenesis through an unknown mechanism [60,61]. β-ionone may cause cancer cell-specific retinoid “overdosing” by potently stimulating vitamin A uptake through STRA6. A related finding is that fenretinide has anti-cancer activity and much fewer side effects than retinoic acids [62], but its mechanism of action is not well understood. Because fenretinide strongly suppresses STRA6-catalyzed retinol loading and stimulates STRA6-catalyzed retinol release [50], fenretinide’s interaction with excessive STRA6 on cancer cells may cause retinoid overloading and contribute to fenretinide’s anti-cancer activity. In addition to cancer treatment, boosting natural vitamin A uptake may have other therapeutic benefits. A related recent finding is that vitamin A and all-trans-retinoic acid synergistically increase retinol uptake and retinyl ester storage in neonatal rat lung [54,63,64,65] and antibody production by the spleen [66]. The ability of exogenous retinoic acid to stimulate STRA6’s retinol uptake activity [50] may contribute to enhanced retinoid uptake. 

## 7. Transmembrane Pathway for Vitamin A Transport by STRA6

**Figure 7:**
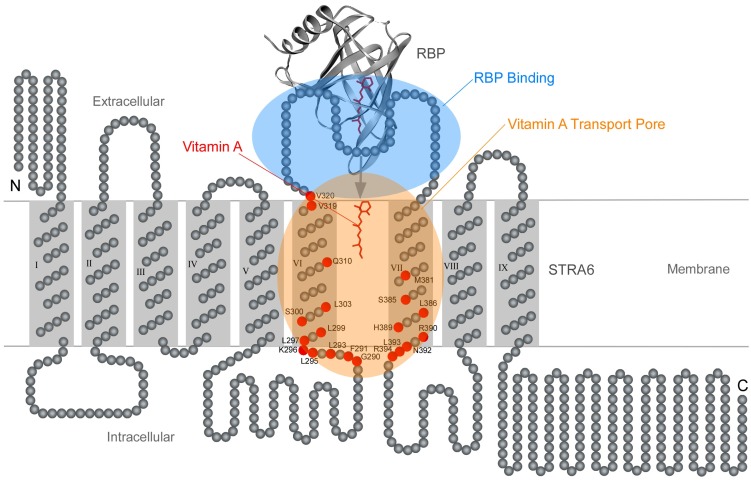
Schematic diagram of the locations of key positions in STRA6 whose acute modifications block vitamin A transport by STRA6. The transmembrane topology model of STRA6 is depicted together with the crystal structure of holo-RBP. STRA6’s key RBP binding domain is shaded in blue. The transmembrane domains and adjacent regions that are known to be part of the vitamin A transport pore are shaded in orange. Residues whose acute modification impedes vitamin A transport by STRA6 are represented as red circles. This figure is based on Figure 9 of reference 67.

STRA6 has nine transmembrane domains, but their functions in vitamin A uptake were completely unknown when the receptor was discovered. A related question to solve is how STRA6 interacts with its transport substrate vitamin A. Membrane proteins that have more than seven transmembrane domains tend to function as transporters or channels. The large number of transmembrane domains allows the formation of a pore, through which the substrate can pass and the transport can be regulated. STRA6 functions as both a receptor for RBP and a transporter to take up vitamin A. Consistently, experimental evidence pointed to the existence of a pore or “passageway” through which vitamin A is transported through STRA6’s transmembrane domains [67]. The key technique that revealed the vitamin A “passageway” in STRA6 is acute chemical modification, which makes it possible to acutely modify a functional protein in a site-specific manner and allows the introduction of a wide variety of side chains that are not possible by mutagenesis alone. By screening chemical reagents of different sizes and charges and systematically scanning the residues in or near transmembrane helices VI and VII of STRA6, many key residues in STRA6 that are aligned in the direct path of vitamin A transport by STRA6 were identified (Figure 7). Acute modification of any of these key residues blocks vitamin A transport by STRA6 and these blocking effects are both highly site specific and chemical modification specific. These results suggest that STRA6’s functions go beyond binding RBP and releasing vitamin A from RBP. Although free vitamin A can diffuse through membranes by itself, vitamin A released from RBP by STRA6 passes through a defined pathway in the multitransmembrane domains of STRA6. Interestingly, many key residues in STRA6 whose modifications block vitamin A transport are located near the intracellular loops (Figure 7). This suggests that the defined path of vitamin A transport within STRA6 goes as far as the intracellular loops and that STRA6 controls vitamin A transport all the way to the intracellular side. This defined pathway for vitamin A transport within STRA6 provides the possibility of regulating its transport, analogous to the gating of ion channels by controlling their pores [68,69,70,71,72,73,74,75]. How STRA6 transport’s activity is regulated is still unknown and is an interesting topic of future investigation. 

## 8. Comparing RBP/STRA6 Interaction with RBP/TTR Interaction

**Figure 8:**
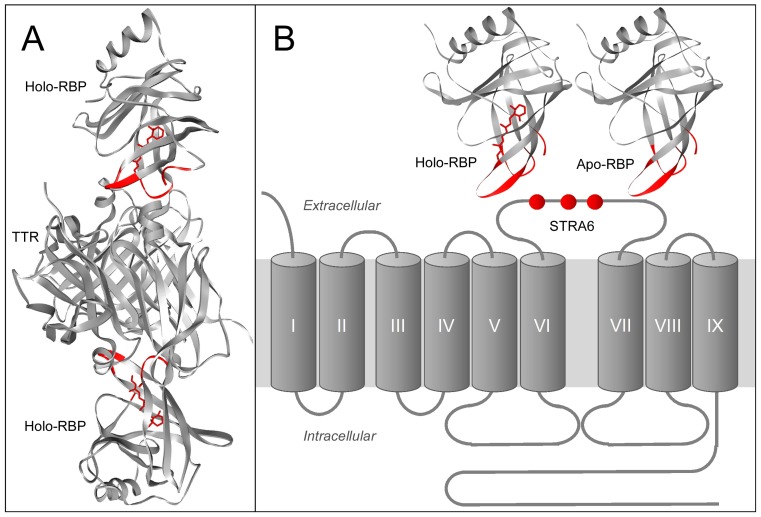
Comparison of RBP’s interaction with TTR and RBP’s interaction with STRA6. Both TTR and STRA6 interact with the vitamin A exit end of RBP. **A.** Crystal structure of RBP/TTR complex. TTR interacts with only holo-RBP, but not apo-RBP. **B.** STRA6 binds to both holo-RBP and apo-RBP. The three residues known to be essential for RBP/STRA6 interaction are labeled in red in the STRA6 topology model. In both A and B, residues located at the vitamin A exit end of RBP are labeled in red.

Holo-RBP in the blood is in complex with the thyroxine binding protein transthyretin (TTR). The holo-RBP/TTR complex increases the molecular weight of holo-RBP and reduces its loss through glomerular filtration in the kidney. The crystal structure of holo-RBP in complex with TTR has been determined [29,76,77]. Because TTR blocks the vitamin A exit end of RBP in its complex with holo-RBP (Figure 8A), TTR makes it even less likely for holo-RBP to randomly release retinol. The much higher binding affinity of TTR/holo-RBP interaction as compared to TTR/apo-RBP interaction is an important mechanism to selectively deplete apo-RBP from the blood through kidney filtration. Drugs that disrupt TTR and holo-RBP interaction lead to the depletion of holo-RBP from blood. In contrast, overwhelming experimental evidence from many independent labs has shown that STRA6 binds both holo-RBP and apo-RBP [33,38,39,78,79] (Figure 8B). This mechanistic difference is one of the most crucial findings regarding STRA6’s vitamin A uptake mechanism. Without the selective depletion of apo-RBP through TTR interaction with holo-RBP, there would be sufficient apo-RBP in the blood to compete with holo-RBP to bind to STRA6. Apo-RBP can interact with STRA6 to deplete cells of their vitamin A store [39,78]. However due to the relatively low amount of apo-RBP as compared to holo-RBP under physiological conditions, STRA6 mediates retinol influx, not retinol efflux, in the serum [78]. 

Before the RBP receptor was identified, it was also known to interact with the vitamin A exit end of the RBP, as summarized previously [80]. Although TTR and STRA6 compete for the binding to RBP, STRA6’s affinity is higher than TTR for RBP [33,79,80]. This was also demonstrated recently through systematic analysis of RBP/STRA6 and RBP/TTR interactions by Chou and colleagues [79]. They determined that the Kd of RBP/TTR interaction is 0.9 μM, but the Kd of RBP/STRA6 interaction is 59 nM [79]. The competition with TTR is a likely driving force for the high affinity binding between RBP and STRA6 despite the fact the holo-RBP concentration in the blood is much higher than the Kd of RBP and STRA6 interaction. Berry *et al*. claimed that TTR completely inhibits holo-RBP’s interaction with STRA6 [81]. Contrary to this claim, STRA6 has no problem in taking up vitamin A from the native holo-RBP/TTR complex [33,38,78]. TTR partially, but not completely, inhibits STRA6’s interaction with holo-RBP [38]. Berry *et al*. did not perform any HPLC analysis or any assay of vitamin A uptake from serum, the natural source of RBP/TTR complex. Using purified native holo-RBP/TTR complex from human serum, it has been shown that STRA6 effectively catalyzes retinol release from this complex and in coupling to LRAT [38]. 

Although RBP/STRA6 interaction has high affinity [33,79], this interaction is also transient, as demonstrated by independent studies [38,79]. The t_1/2_ for RBP release from STRA6 is about 10 min [79]. On one hand, this transient interaction makes physiological sense and is absolutely essential for continuous vitamin A uptake by the RBP receptor. Each RBP molecule binds to one vitamin A molecule. If RBP remains permanently bound to STRA6, the permanent binding would prevent the next RBP from delivering the next vitamin A molecule. On the other hand, the time it takes RBP to be released from STRA6 likely determines how fast vitamin A can be continuously transported into the cell. Even t_1/2_ of 10 min is much slower than channels and transporters that transport free ligands. However, there is a limit on how fast RBP can be released by STRA6 given the high affinity interaction between these two proteins, as described above. Since K_d_ is determined by the ratio of K_off_ and K_on_, there is likely a limit on how evolution can finely tune the RBP/STRA6 interaction to improve K_on_ and K_off_. 

## 9. RBP-Dependent Pathway and RBP-Independent Pathway

RBP knockout mice have largely vision-specific phenotypes under vitamin A sufficient conditions, but RBP is essential in mobilizing the liver-stored vitamin A [82]. There are two puzzles related to this early finding. Is the vast quantity of liver-stored vitamin A (which only RPB can mobilize) only used by the eye? What can compensate for the lack of RBP? Quadro and colleagues showed that retinyl esters bound to lipoproteins can partly compensate for the lack of RBP under vitamin A sufficient conditions [28,83]. Retinyl ester bound to lipoprotein depends on immediate vitamin A intake from food because its source is the small intestine, not the liver-stored vitamin A [82,84]. Under vitamin A deficient conditions that mimic the natural environment and limit the generation of retinyl esters bound to lipoproteins, the RBP knockout has the systemic phenotype of embryonic lethality [28,83]. Consistently, in mouse embryo culture, where yolk-sac is the source of vitamin A and there is no retinyl ester pathway, knockdown of RBP expression in the yolk sac causes severe developmental defects [85]. These studies demonstrated that knockout mice may miss important functions of a protein due to complex environmental or genetic contributions from other factors. 

The retinyl ester pathway, which is the major RBP-independent mechanism of vitamin A delivery, has been known for almost 40 years [86]. The RBP pathway and the retinyl ester pathway differ in many aspects. First, blood retinol/RBP levels, but not retinyl esters, are homeostatically regulated [87]. Second, the retinyl ester pathway depends on immediate presence of vitamin A in food and cannot be relied on during vitamin A deficiency, which is common in natural environments [25]. Third, the uptake mechanism for retinol bound to RBP is also different from the uptake of retinyl esters bound to lipoproteins, which use the uptake mechanisms for cholesterol and other lipids. A wide variety of molecules participate in the uptake of lipoproteins including the LDL receptor [88], LRP-I [89], VLDLR [90], heparin sulfate proteoglycan [91] and lipoprotein lipase [91,92,93]. The wide expression of these proteins suggests that retinyl esters bound to lipoproteins will be taken up by a much wider array of cell types than those that specialize in vitamin A uptake from RBP. Fourth, the retinyl ester pathway is associated with toxicity due to its lack of homeostatic control and its association of ubiquitous lipid uptake pathways. Both animal and human studies found that more toxicity is associated with vitamin A delivery independent of RBP [86,94]. An excessive dose of vitamin A is toxic only when vitamin A in the circulation is presented to cells in a form other than bound to RBP, such as in retinyl esters [23]. 

## 10. Phenotypic Variability Caused by Mutations in RBP or STRA6

Complete vitamin A deficiency is lethal in vertebrates. However, mutations in most retinoid-related proteins do not cause lethal phenotypes due to functional redundancy. STRA6 mutations cause a wide spectrum of pathological phenotypes including anophthalmia, mental retardation, congenital heart defects, lung hyperplasia, intrauterine growth retardation, and embryonic lethality [95,96,97,98,99,100,101,102]. STRA6 knockout has more severe developmental defects in humans than most other retinoid-related proteins [11]. The severe phenotypes caused by STRA6 mutations suggested that there may be less redundancy in vitamin A transport mechanisms than other aspects of retinoid signaling in humans (species difference will be discussed below). Redundancy is necessary to ensure that backup mechanisms take over when one mechanism fails. 

Mutations in a ligand and its receptor do not necessarily generate identical phenotypes. For example, a human embryo without the RBP receptor can be directly impacted by the loss of placental absorption of vitamin A from maternal RBP. In contrast, a human embryo without RBP still has the functional RBP receptor and functional maternal RBP for vitamin A delivery to the embryo through the placenta. The first human RBP mutations identified led to partial loss of RBP function and showed a vision-specific phenotype of RPE dystrophy at a young age [103,104]. RPE is essential for vision, especially in the maintenance and support of photoreceptor cells [105,106]. Although human STRA6 mutations can cause anophthalmia, human RBP mutations were previously not known to cause anophthalmia. A recent breakthrough study by Chou and colleagues discovered human RBP mutations that cause anophthalmia and a new mode of maternal inheritance [79]. They found that the pathological phenotypes are preferentially transmitted if the mutations are inherited from the mother. This new mode of maternal inheritance can be explained by the roles of RBP and STRA6 in the placenta’s vitamin A transport to the fetus [79,107]. Mechanistic studies revealed that these mutant RBPs block vitamin A transport by STRA6 in a precise and dominant manner [79]. These mutant RBPs bind vitamin A loosely and tend to lose their vitamin A cargo in a receptor-independent manner. Most importantly, they have an affinity for STRA6 that is 30–40 fold higher than that of the wild-type RBP. These properties lead to the blockage of STRA6 by the mutant RBP in a dominant negative manner. Although the blood contains about 66% wild-type RBP, the blocking effect of mutant RBP is sufficient to cause developmental defects like anophthalmia. 

Phenotypes caused by STRA6 mutations in humans are highly variable, ranging from embryonic lethality to “mild” eye-specific phenotypes, although the eye phenotype is shared by all patients surviving to birth [95,96,97,98,99,100,101,102]. The variability in phenotypes is likely caused by variable degrees in the loss of STRA6 function (genetic factor) and the variability in vitamin A intake of the affected individuals (environmental factor). Vitamin A intake alone (either insufficient or excessive) is sufficient to cause severe developmental defects without any genetic contribution. RBP/STRA6 independent mechanisms of retinoid delivery (e.g., the retinyl ester pathway if retinoid intake is sufficiently large) may partly compensate the absence of RBP or STRA6. But the severe phenotypes caused by the loss of these proteins showed that complete dependence on the RBP-independent pathway is highly risky. Without the RBP/STRA6 pathway, an individual is at the “mercy” of the environment by completely depending on constant but not excessive dietary vitamin A intake. 

## 11. Points of Potential Confusion

**(1) Species differences:** In vitamin biology, species difference can be surprising and confusing. For example, vitamin C is essential for human survival, but vitamin C is not a vitamin for mouse [108]. This sharp contrast is likely a result of the diet difference between human ancestors (which relied on vitamin C-rich food) and rodent ancestors (which survived on food poor in vitamin C). Species difference between mouse and human in vitamin A biology is less extreme but is still highly significant. Loss of the ALDH1A3 gene causes anophthalmia in human [109,110], but not in mice [111]. Mutations in the gene for RARβ also cause anophthalmia in human [112], but RARβ knockout alone does not cause this phenotype in mice [113]. ABCA4 is a transporter that transports a vitamin A derivative in vertebrate photoreceptor cells [114,115,116,117]. Complete loss of ABCA4 causes severe photoreceptor degeneration (retinitis pigmentosa) in human [118,119]. However, ABCA4 knockout alone is not sufficient to cause significant photoreceptor degeneration in mouse and it needs to be combined with RDH8 knockout to generate severe degeneration phenotypes [115,120]. If the mouse model is the only source of information, it may be concluded that ALDH1A3 and RARβ play no role in anophthalmia and that ABCA4 plays no role in retinitis pigmentosa. Similarly, STRA6 mutations cause severe developmental defects in humans, but STRA6 knockout mice, like RBP knockout mice, have only vision-specific phenotypes of blindness due to the lack of vitamin A [52,121]. 

There are many possible reasons that can explain the species differences between mouse and human. For example, lab mice are routinely fed with highly vitamin A-enriched diet, as compared to natural diets for animals or humans. The highly vitamin A-enriched diet promotes the retinyl ester pathway of vitamin A delivery. Mouse is also known to be much more tolerant of retinoid toxicity than human. For example, rodents are about 100 times less sensitive to isotretinoin’s teratogenic effect than human [122]. The initial approval of isotretinoin for clinical use in human without the knowledge of its teratogenic effect was at least partly due to the insensitivity of rodent models. Consistent with the importance of diet in influencing knockout phenotypes, RBP knockout mice have vision-specific phenotypes when fed with standard laboratory diet, but have severe developmental defects under vitamin A deficient conditions that mimic the natural environment [83]. Interestingly, loss of STRA6 in zebrafish causes highly suppressed tissue vitamin A uptake and severe developmental defects [39]. The reason that zebrafish is better than mouse in recapitulating human phenotypes is likely because of its less dependence on the retinyl ester pathway and its lack of highly vitamin A-enriched food. 

**(2) Apo-RBP, holo-RBP, and insulin resistance:** In 2005, elevated RBP level was identified as a potential signal that triggers insulin resistance [123]. The receptor that senses RBP to trigger insulin resistance is expected to sense RBP concentration change in the micromolar range, given the fact that blood RBP concentration is about 2.6 μM in healthy people [124]. Whether STRA6, which binds to RBP at nanomolar affinity [33,79], is involved in sensing RPB to cause insulin resistance has been discussed in detail previously [125,126] and is only briefly discussed here. One group of authors (Berry *et al*.) hypothesized that STRA6 binds RBP to take up vitamin A and cause insulin resistance at the same time [127]. They claimed that the STRA6 binds only holo-RBP, not apo-RBP, and that holo-RBP’s binding to STRA6 causes insulin resistance [128]. All these claims contradict independent studies from many other groups. First, the model by Berry *et al*. failed to explain why the micromolar concentrations of holo-RBP in healthy people’s blood do not cause insulin resistance. Second, obesity is associated with significantly higher apo-RBP, but normal holo-RBP [124], and that RBP-to-retinol ratio is elevated in type 2 diabetes [129]. Third, Motani and colleagues developed a drug that specially lowers holo-RBP concentration by converting it to apo-RBP, but this this drug does not prevent insulin resistance [130]. Fenretinide was previously thought to lower insulin resistance by lowering RBP, but was later found to act through an RBP-independent mechanism [131]. Fourth, Norseen and colleagues showed that apo-RBP is much more potent than holo-RBP in initiating signal transduction changes [132]. Fifth, independent studies by Farjo and colleagues and Norseen and colleagues showed that RBP’s effect on insulin resistance is independent of STRA6 [132,133]. Sixth, overwhelming evidence showed that apo-RBP does bind to STRA6. It has been demonstrated directly that both apo-RBP and holo-RBP binds to STRA6 [33,41]. Both Isken and colleagues [39] and we [41] showed that apo-RBP can promote retinol efflux through STRA6. In addition, Chou and colleagues performed extensive analysis of the kinetics of apo-RBP and holo-RBP binding to STRA6 in their discovery of human RBP mutants that block STRA6’s vitamin A uptake activity [79]. They also found high affinity binding of apo-RBP to STRA6 (Kd = 59 nM). 

Major technical problems and other issues can explain the contradiction between the studies by Berry *et al*. and studies by other groups [125,126]. For example, Berry *et al*. depended on a commercial antibody to link their study to STRA6 [127]. However, this antibody that they used to purify or detect human STRA6 does not recognize human STRA6. First, there is no theoretical reason to suggest that this antibody should recognize human STRA6. The mouse peptide used in the immunization is almost completely different from the human counterpart. Second, two independent groups have demonstrated that this antibody does not recognize human STRA6 [78,134]. Consistently, the intensities of so-called “STRA6” bands never increased after STRA6 transfection in all figures [127]. The true identities of these “STRA6” bands are still unknown [127]. It has been well established from studies in the past decades including crystallographic studies that retinol forms a 1:1 complex with RBP (the definition of holo-RBP). However, Berry *et al*. performed ^3^H-retinol uptake assays by acutely mixing free ^3^H-retinol with unlabeled holo-RBP and applying this mixture without removing free ^3^H-retinol [128]. Because ^3^H-retinol/RBP was not properly formed and no free ^3^H-retinol was removed, this assay is a free ^3^H-retinol diffusion assay, not an assay of STRA6-mediated ^3^H-retinol uptake from ^3^H-retinol/RBP complex. In addition, Berry *et al*. exclusively used RBP made in bacteria and never used mammalian cell-produced RBP or RBP purified from the blood, as in numerous other studies of RBP. Their signal transduction assays lack negative controls to show that the events were not due to bacterial contamination, which is sufficient to cause signal transduction changes (e.g., through pattern recognition receptors).

## 12. Conclusion

Evolution’s commitment to specific vitamin A delivery is evident from the fact that a whole RBP protein is produced to transport just one vitamin A molecule, and RBP is the principle carrier of vitamin A in the blood even though most retinoids have the ability to diffuse systemically without any carrier protein. The likely driving forces for the evolution of the precise, sustained, and efficient system of vitamin A transport include the need for all vertebrates to survive vitamin A deficiency (which is common in the natural environments), the high demand of certain organs for vitamin A (e.g., the eye), and the short-term and long-term toxicity associated with random retinoid diffusion [135]. If vitamin A is considered a drug that all vertebrates need to take to survive, the RBP/STRA6 system is a finely tuned “drug delivery system” that combines sustained release, specific targeting and controlled delivery. Under normal physiological conditions, RBP mobilizes the liver-stored form of vitamin A to maintain a stable blood concentration of vitamin A without a need for constant intake (“sustained release”). Without RBP, the vast amount of vitamin A stored in the liver cannot be used by other organs. During vitamin A deficiency when blood RBP levels drop, the high-affinity interaction between RBP and STRA6 provides a certain degree of buffering to ensure efficient vitamin A uptake even in the presence of low RBP concentration (the Kd of STRA6/RBP interaction is much lower than the micromolar physiological concentrations of RBP [33]). The highly specific interaction between RBP and STRA6 ensures precise targeting of RBP to cells and organs that need vitamin A for proper functioning (“specific targeting”). The mechanism used by STRA6 to take up vitamin A ensures that the target cells only take up vitamin A when they have a means to store it, and prevents excessive uptake and random diffusion of vitamin A (“controlled delivery”). STRA6’s vitamin A uptake mechanism is distinct from known membrane transport mechanisms such as active transport, channels and facilitated transport [38]. The new membrane transport mechanism mediated by STRA6 results from its diverse catalytic activities including the catalysis of retinol release from holo-RBP, retinol loading into apo-RBP, retinol exchange between retinol binding proteins. 

Elucidation of the transport mechanism of STRA6 can both facilitate the development of small molecule-based drugs that target this new type of cell-surface receptor and help to understand the action of existing drugs. STRA6’s vitamin A uptake activity can be strongly stimulated by many compounds with the β-ionone ring [38,50]. This strategy may be employed to treat human diseases caused by decreased tissue retinoid levels without using a retinoid (e.g., skin diseases and cancer). Fenretinide, an anticancer agent and an agent that can decrease serum RBP level, strongly promotes retinol release activity of STRA6 [50]. Since STRA6 is highly overexpressed in cancer cells, fenretinide’s interaction with STRA6 in cancer cells may cause retinoid overloading and contribute to fenretinide’s anti-cancer activity. Fenretinide’s dependence on STRA6 in cancer treatment was demonstrated in a recent study [53]. Fenretinide also depends on STRA6 to release retinol from RBP, as demonstrated by recent mechanistic experiments [50,52]. 

There are still unsolved and intriguing questions in the field. Examples are listed below: 

**(1) STRA6’s roles in different organs:** Because STRA6 knockout does not cause anophthalmia in mouse as in human, this is an opportunity to mechanistically study STRA6’s role in adult vision [52,121]. STRA6 knockout leads to the loss of most of its vitamin A store for vision and the degeneration of cone photoreceptor cells, which are essential for daylight vision and color vision [52,121]. STRA6 is also expressed in many other vertebrate organs [80], but its roles are less well understood and are just starting to emerge. Recently, how STRA6’s vitamin A uptake activity contributes to adipogenesis [134] and human epidermal differentiation [136,137] was investigated. Muenzner and colleagues identified the role of STRA6’s vitamin A transport activity in controlling the differentiation of adipocyte progenitor cells [134]. Skazik and colleagues used 3D organotypic human skin model to uncover the roles of STRA6 in regulating vitamin A-mediated differentiation and proliferation of human skin [136,137]. These new studies using different model systems have begun to reveal important functions of this vitamin A uptake receptor/transporter in diverse physiological contexts in different species. 

**(2) STRA6 regulation:** STRA6/CRBP-I-mediated uptake can naturally saturate due to the role of CRBP-I as a retinol binding protein (one CRBP-I molecule only binds one vitamin A molecule). However, how STRA6/LRAT-mediated retinyl ester formation is regulated, is unknown. Cells that express both STRA6 and LRAT would be bloated with retinyl esters in days if not months if there was no regulatory mechanism to regulate the uptake. How cellular uptake of vitamin A through STRA6 is regulated to prevent excessive accumulation of retinyl esters is still unknown. Regulation may occur at the protein or mRNA level. The transcriptional regulation of STRA6 mRNA in several organs has been investigated in several recent studies, which revealed dynamic regulation of its transcription [40,54,66,138,139,140].

**(3) Interaction of RBP-dependent and RBP-independent pathways:** Cellular uptake of retinyl esters can partially compensate for the loss of RBP during vitamin A sufficient or excessive conditions. Do the retinyl ester uptake pathway and the RBP pathway mutually influence each other? Do proteins involved in retinol uptake through STRA6 (e.g., CRBP-I or LRAT) also contribute to retinyl ester uptake? Since retinyl ester uptake is associated with uptake of cholesterol and other lipids, is the uptake process regulated through cholesterol feedback regulation [141]? 

**(4) STRA6 structures:** The crystal structures of RBP and RBP/TTR complex were determined 20 or 30 years ago [76,77,142]. The crystal structure of STRA6 or its complex with RBP is currently unknown. A large-scale purification of STRA6 protein has been achieved recently [143]. The existence of the super receptor binding RBP mutants may facilitate the determination of the complex between RBP and STRA6 given the 30–40 fold increase in affinity [79]. The rapid advances of X-ray crystallography techniques and cryo-EM techniques may help to solve the 3D structure of this new type of transmembrane receptor in the future. 
